# Microhemorrhages in the corpus callosum after treatment with extracorporeal membrane oxygenation

**DOI:** 10.1186/cc14357

**Published:** 2015-03-16

**Authors:** S Riech, P Hellen, O Moerer, K Kallenberg, M Müller, M Quintel, M Knauth

**Affiliations:** 1 University Medical Center Goettingen, Germany

## Introduction

Cerebral microhemorrhages (MH) are diminutive focal bleedings which can be detected best by MRI using susceptibility-weighted imaging sequences (SWI). They can be found in a variety of neurologic diseases. The pattern of distribution can lead to the underlying pathomechanism [1]. Survivors of high-altitude cerebral edema (HACE) showed multiple MH, predominantly in the splenium of the corpus callosum. Mountaineers with a lack of acclimatization to high altitudes tend to suffer from HACE. Hypoxemia in great heights is discussed to be the main trigger of HACE [2]. Acute respiratory distress syndrome (ADRS) is characterized by oxygenation failure in mechanically ventilated patients. The severity is classified by the ratio of arterial oxygen tension to fraction of inspired oxygen [3]. In some patients suffering from severe ARDS, refractory to conventional therapy, venovenous extracorporeal membrane oxygenation therapy is the therapeutic option to ensure oxygenation.

## Methods

Retrospectively, we examined 20 patients with cerebral MRI (including SWI) who had suffered from severe ARDS and received ECMO therapy. The MRI slides were anonymized and analyzed by two experienced neuroradiologists. Based on the distribution pattern and characteristic, a modified HACE score (mHCS) was surveyed [2].

## Results

Six of 20 patients (30%) showed multiple MH with emphasis in the splenium of the corpus callosum. Eight patients had sporadic MH in the parenchyma of the brain but not in the corpus callosum. The remaining six patients had no intracerebral alterations. The distribution of MH with involvement of the splenium resembled that seen in HACE survivors.

## Conclusion

Based on these results, we postulate that hypoxemia is one of the main players in the development of splenium-associated MH, not only in HACE but also in severe ARDS and other diseases accompanied with severe hypoxemia. Further investigations have to examine potential triggers and special circumstances concerning ARDS treatment which lead to MH in this distinctive pattern.
